# Reduced brain activation during inhibitory control in children with *COMT* Val/Val genotype

**DOI:** 10.1002/brb3.577

**Published:** 2016-10-05

**Authors:** Lora M. Cope, Jillian E. Hardee, Mary E. Soules, Margit Burmeister, Robert A. Zucker, Mary M. Heitzeg

**Affiliations:** ^1^ Department of PsychiatryUniversity of MichiganAnn ArborMIUSA; ^2^ Addiction CenterUniversity of MichiganAnn ArborMIUSA; ^3^ Molecular & Behavioral Neuroscience InstituteUniversity of MichiganAnn ArborMIUSA; ^4^ Department of Human GeneticsUniversity of MichiganAnn ArborMIUSA

**Keywords:** catechol *O*‐methyltransferase, children, dopamine, functional magnetic resonance imaging, go/no‐go, inferior frontal gyrus, insula, putamen, response inhibition, substance use disorder risk

## Abstract

**Introduction:**

Behavioral undercontrol is a well‐established risk factor for substance use disorder, identifiable at an early age well before the onset of substance use. However, the biological mechanistic structure underlying the behavioral undercontrol/substance use relationship is not well understood. The enzyme catechol *O*‐methyltransferase (COMT) catabolizes dopamine and norepinephrine in the prefrontal cortex and striatum, brain regions involved in behavioral control. The goal of this work was to investigate the association between genetic variation in COMT functioning and fronto‐striatal brain functioning during successful inhibitory control, a critical aspect of behavioral control.

**Methods:**

Participants were 65 (22 female) 7–12 year olds who were genotyped for the functional *COMT* Val^158^Met (rs4680) single‐nucleotide polymorphism and underwent functional magnetic resonance imaging while performing a go/no‐go task. The majority of the sample (80%) had at least one parent with a history of alcohol use disorder and were thus at heightened risk for substance use disorders.

**Results:**

There was a significant main effect of genotype on brain activation in left and right putamen during successful versus failed inhibition and in right inferior frontal gyrus/insula during successful inhibition versus baseline. Follow‐up tests revealed that Met homozygotes had greater activation in each region relative to Val homozygotes.

**Conclusions:**

These results are relevant for understanding how specific genes influence brain functioning related to underlying risk factors for substance use disorders and other disinhibitory psychopathologies.

## 1 Introduction

Substance use initiation by age 13 is associated with greater risk of developing a substance use disorder (SUD) (Grant & Dawson, 1997) and can have a negative impact on academic achievement, family and peer relationships, and psychosocial maturation (Schulenberg, Bryant, & O'Malley, 2004). Understanding the biological risk structure that drives early substance use onset may aid in the development of more effective prevention strategies to reduce the incidence and impact of SUD.

One of the most robust risk factors for SUD, identifiable prior to the initiation of substance use, is behavioral undercontrol (McGue, Iacono, Legrand, Malone, & Elkins, 2001; Zucker, Heitzeg, & Nigg, 2011). Poor inhibitory control has been proposed as an underlying cognitive mechanism contributing to behavioral undercontrol (Zucker et al., 2011). At the neural level, the mesolimbic dopaminergic pathway is fundamentally related to these operations (Dalley, Mar, Economidou, & Robbins, 2008; Diergaarde et al., 2008). The dopamine system undergoes dramatic change during adolescence, concomitant with substance use initiation and escalation (Luciana, Wahlstrom, Porter, & Collins, 2012; Spear, 2011). It is also centrally related to the reinforcement potential of drugs of abuse (Everitt & Robbins, 2005; Robinson & Berridge, 2000), and has been implicated in pre‐existing vulnerability to addiction (McBride & Li, 1998; Volkow et al., 2002). An emerging literature describes the impact of dopaminergic genetic variation on brain functioning during inhibitory control in healthy young adults (Cummins et al., 2012) and typically developing adolescents (Braet et al., 2011).

An important modulator of dopamine activity in the prefrontal cortex and striatum is the enzyme catechol *O*‐methyltransferase, which catabolizes dopamine and norepinephrine (Chen et al., 2004). A functional valine (Val) to methionine (Met) substitution at codon 158 in the gene that codes for catechol *O*‐methyltransferase (*COMT* Val^158^Met; rs4680) results in a 3–4 fold enzymatic activity increase and concomitant synaptic dopamine reduction in individuals homozygous for the Val allele (Chen et al., 2004). Studies of healthy youth (ages 8–14) have found the *COMT* Met allele to be associated with better inhibitory control (Diamond, Briand, Fossella, & Gehlbach, 2004), particularly in males (Barnett et al., 2007). A recent large‐scale study of healthy adolescents (mean age 14.4; White et al., 2014) found a *COMT *× sex interaction in the presupplementary motor area during stop‐signal inhibitory control, with male Val homozygotes showing the highest brain activity relative to the other two male genotypes; in females, however, this pattern was not observed, supporting other evidence that sex moderates the effects of *COMT* on brain activity (reviewed in Harrison & Tunbridge, 2008).

This study sought to better understand the association between the *COMT* Val^158^Met polymorphism and brain activity during inhibitory control prior to significant substance use in males and females at high risk for SUD (*N *=* *65). Participants were 7–12 years old with minimal history of substance use. Based on previous work (Barnett et al., 2007; Diamond et al., 2004), we expected to find better inhibitory control performance among those with at least one Met allele as well as greater brain activity during inhibitory control in frontal and striatal areas among Val homozygotes. In light of sex‐related findings from prior studies, we also expected effects to be stronger in males than in females.

## 2 Material and methods

### 2.1 Participants

Participants were 65 right‐handed youth^1^ (22 female), aged 7.8–12.9 years at the time of the fMRI scan (*M *=* *10.4, *SD *= 1.17). They were recruited from the Michigan Longitudinal Study (MLS; Zucker, Ellis, Fitzgerald, Bingham, & Sander, 1996; Zucker et al., 2000), an ongoing, multiwave study of community‐recruited families with and without parental alcohol use disorder. Eighty percent of participants in the current sample had at least one parent with an alcohol use disorder. MLS assessments are conducted every three years starting when the children are age 3–5; adolescents and young adults are also assessed every year from age 11–26. Families were excluded during MLS recruitment if the target child displayed evidence of fetal alcohol effects or the mother reported drinking during pregnancy. Exclusionary fetal alcohol characteristics included prenatal or postnatal growth retardation or both, central nervous system involvement, and characteristic facial dysmorphology (Loukas, Fitzgerald, Zucker, & von Eye, 2001; Sokol & Clarren, 1989). Full details on assessment and data collection in the MLS can be found elsewhere (Zucker et al., 2000).

For this study, participants were excluded if they had: neurological, acute, or chronic medical illness; current, active Axis I disorder (not including past mood disorder or current or past anxiety disorder, conduct disorder, or ADHD); current or recent (within 6 months) treatment with centrally active medications; MRI contraindications such as metal implants or claustrophobia; IQ <70; or history of psychosis in first‐degree relatives. Participants who were taking medication for ADHD were asked to abstain at least 48 hr before the MRI scan. See Table 1 for demographic and psychometric variables.

**Table 1:** Demographic and psychometric variables

	Met/Met	Val/Met	Val/Val	Total Sample	Test
*n*	11	34	20	65	
Demographic data					
Sex (M/F)	8/3	22/12	13/7	43/22	*p *=* *.940^a^
Age at Scan: Mean (SD)	10.4 (1.08)	10.2 (1.22)	10.6 (1.14)	10.4 (1.17)	*F*(2, 62) = 0.73*p *=* *.485
Race and Ethnicity (%)					
Caucasian	13.8	27.7	18.5	60.0	*p *=* *.124^a^
Hispanic	0.0	10.8	0.0	10.8	
African American	1.5	10.8	6.2	18.5	
Biracial	1.5	3.1	6.2	10.8	
Parental AUD (FH+/FH−)					
Ever	8/3	26/8	18/2	52/13	*p *=* *.377^a^
In Child's Lifetime	6/5	22/12	13/7	41/24	*p *=* *.883^a^
Substance Use^b^ (Yes/No)	4/6	8/26	6/14	18/46	*p *=* *.554^a^
IQ: Mean (SD)^c^	101.7 (9.70)	102.4 (12.02)	103.8 (16.00)	102.7 (12.93)	*F*(2, 59) = 0.11*p *=* *.897
Symptomology: Mean (SD)					
CBCL Externalizing	6.6 (5.80)	10.5 (8.91)	7.0 (5.09)	8.7 (7.56)	*F*(2, 62) = 1.93*p *=* *.154
CBCL Internalizing	3.8 (3.07)	7.7 (6.94)	5.3 (3.83)	6.3 (5.75)	*F*(2, 62) = 2.37*p *=* *.102
DSM‐IV lifetime diagnosis (count)					
ADHD, any type	0	5	3	8	*p *=* *.536^a^
Generalized Anxiety Disorder	0	0	0	0	–
Major Depression Disorder	0	0	0	0	–
Oppositional Defiant Disorder	1	4	2	7	*p *=* *1.000^a^
Conduct Disorder	0	0	1	1	*p *=* *.477^a^
Motion parameters: Mean (SD)					
Translation (mm)^d^	0.046 (0.027)	0.035 (0.018)	0.031 (0.013)	0.036 (0.019)	*F*(2, 62) = 2.33*p *=* *.106
Rotation (degrees)^d^	0.053 (0.031)	0.039 (0.021)	0.035 (0.016)	0.040 (0.022)	*F*(2, 62) = 2.46*p *=* *.094
Runs excluded	0.09 (0.30)	0.18 (0.46)	0.40 (0.82)	0.23 (0.58)	*F*(2, 62) = 1.33*p *=* *.271

SD, standard deviation; AUD, alcohol use disorder; FH+, alcohol use disorder in one or both parents; FH−, alcohol use disorder in neither parent; CBCL, Child Behavior Checklist (Achenbach & Rescorla, 2001); DSM‐IV, Diagnostic and Statistical Manual of Mental Disorders IV (APA, 1994); ADHD, attention deficit hyperactivity disorder; mm, millimeters.

a Fisher's exact test.

b One subject was missing drinking and drug history scores (Met/Met).

c Three subjects were missing IQ scores (1 Met/Met, 2 Val/Met).

d Defined as the mean difference from one volume to the next.

Parents/guardians of participants provided written informed consent and participants provided written informed assent. Study materials and procedures were approved by the University of Michigan Medical School Institutional Review Board.

### 2.2 Measures

#### 2.2.1 Genotypes

*COMT* Val^158^Met (rs4680) was genotyped by a 5' exonuclease allelic discrimination TaqMan assay, provided by Applied Biosystems from the Drug Metabolism panel (Life Technologies, Grand Island, NY, USA) and allelic discrimination analysis was performed using the software SDS v2.2.2 (Applied Biosystems, Foster City, CA, USA). This SNP is part of the Illumina addiction biology SNP array designed by Hodgkinson et al. (2008). The panel includes SNPs from 130 candidate genes for alcoholism, addictions, and disorders of mood and anxiety and is genotyped using the Illumina GoldenGate platform. About half of the larger overall MLS sample was genotyped by both the Taqman assay and the Illumina Addiction panel, and no discrepancies were observed in >200 samples. There was no significant deviation from Hardy–Weinberg equilibrium in either the overall or the fMRI subsample.

#### 2.2.2 Psychometric measures

The school age Child Behavior Checklist (CBCL; Achenbach & Rescorla, 2001) was used to assess externalizing (e.g., aggressive, rule‐breaking) and internalizing (e.g., anxious, depressive) symptomology, as reported by parents. The Wechsler Intelligence Scale for Children (WISC‐III; Wechsler, 1991) was used to assess full‐scale IQ. Family history of AUD was defined as follows: A subject was considered *family history positive* (FH+) if one or both parents ever had a diagnosis of alcohol abuse or dependence, according to Diagnostic and Statistical Manual of Mental Disorders, 4th Edition (DSM‐IV; American Psychiatric Association, 1994) criteria; if neither parent met these criteria the subject was considered *family history negative* (FH−). In addition, family history of AUD during the child's lifetime was also specified. For diagnoses (e.g., ADHD, major depressive disorder, conduct disorder), the computerized Diagnostic Interview Schedule for Children (C‐DISC; Shaffer, Fisher, Lucas, Dulcan, & Schwab‐Stone, 2000) was given and diagnoses were tallied based on DSM‐IV criteria.

Substance use was assessed every three years between ages 6 and 10 with a Health and Daily Living Questionnaire as part of the regular MLS assessment schedule. Questions covered use of marijuana, alcohol (more than a sip), cigarettes, and other drugs. If relevant, the age at which use occurred and the quantity/frequency of use was recorded. Subsequent annual assessments (i.e., starting at age 11) involved the Drinking and Drug History Form for Children (Zucker & Fitzgerald, 1994), which also covers age of use as well as quantity and frequency of alcohol, marijuana, nicotine, and other drug use. For the purposes of describing this study sample, substance use was dichotomized (yes/no).

### 2.3 Stimuli and task

A go/no‐go task (Durston, Thomas, Worden, Yang, & Casey, 2002; Hardee et al., 2014; Heitzeg, Nigg, Yau, Zucker, & Zubieta, 2010; Heitzeg et al., 2014) was used to probe response inhibition, or the ability to suppress a prepotent response. Participants were instructed to respond with a button press to target stimuli (all letters except “X”; *p *= .75) and to withhold the button press to nontarget stimuli (“X”; *p* = .25). Stimulus duration was 500 ms with an inter‐stimulus interval of 3500 ms, during which a black screen with a white fixation cross was displayed. All responses that occurred within 3000 ms after stimulus onset were counted. A rapid mixed‐trial event‐related design was used. Participants completed 5 runs, each having 49 trials and lasting 3.5 min. Rates of false alarms (pressing the button for a nontarget stimulus; FAs), hits (pressing the button for a target stimulus), misses (not pressing the button for a target stimulus), and correct rejections (not pressing the button for a nontarget stimulus; CRs) were recorded. Reaction times (measured from the beginning of stimulus presentation) to FAs and hits were also recorded. Finally, a measure of sensitivity, *d*', was calculated as *z*(hit) – *z*(false alarm). Because the corresponding *z*‐score is +∞ when hit rate is 1.0 (i.e., 100%), it is common practice to recalculate hit rate as 1 – 1/(2*N*), where *N* is the number of target stimuli (Macmillan & Kaplan, 1985; Stanislaw & Todorov, 1999). This was done for the five participants with hit rates of 1.0 (3 female, 2 male; 1 Met/Met, 1 Val/Met, 3 Val/Val). See Table 2 for task performance measures.

**Table 2:** go/no‐go task performance measures

	Met/Met		Val/Met		Val/Val	
	M	F	M	F	M	F
Hits (%)	91.58 (13.78)	89.73 (2.39)	93.30 (10.97)	95.82 (2.87)	95.82 (4.60)	97.30 (3.22)
Hit RT (ms)	452.51 (48.82)	508.83 (28.69)	495.82 (103.29)	582.36 (255.58)	483.62 (109.08)	562.48 (90.61)
False Alarms (%)	57.40 (19.41)	30.57 (16.73)	48.34 (19.00)	42.63 (19.21)	50.38 (17.14)	29.54 (21.86)
False Alarm RT (ms)	436.35 (71.23)	459.97 (22.22)	443.00 (75.57)	499.44 (167.81)	435.15 (91.80)	488.11 (71.00)
*d*'	1.54 (0.66)	1.83 (0.62)	1.84 (0.83)	2.08 (0.96)	1.93 (0.65)	2.80 (0.99)

M, male; F, female; RT, reaction time; ms, milliseconds; *d*' is a measure of sensitivity and is calculated as *z*(hit) – *z*(false alarm).

Means with standard deviations in parentheses.

### 2.4 fMRI data acquisition and statistical analysis

Whole‐brain blood oxygen level‐dependent (BOLD) functional images were acquired on a 3.0T GE Signa scanner (Milwaukee, WI, USA) using T2*‐weighted single‐shot combined spiral in/out sequences (Glover & Law, 2001) (TR 2000 ms, TE 30 ms, flip angle 90 degrees, field‐of‐view 200 mm, matrix size 64 × 64, slice thickness 4 mm, 29 slices). High‐resolution anatomical T1 scans were also obtained for spatial normalization. Motion was minimized with foam padding around the head and instructing participants on the importance of keeping still.

Functional images were reconstructed using an iterative algorithm (Noll, Fessler, & Sutton, 2005; Sutton, Noll, & Fessler, 2003) and motion corrected using FSL v5.0.2.2 (FMRIB, Oxford, UK). Runs exceeding 3 mm translation or 3° rotation were excluded^2^. Image preprocessing was completed using Statistical Parametric Mapping (SPM8 [RRID:SCR_007037]; Wellcome Institute of Cognitive Neurology, Oxford, UK). Functional images were spatially normalized to the Montreal Neurological Institute (MNI) template and smoothed with a 6 mm full‐width at half‐maximum (FWHM) smoothing kernel. Low‐frequency noise was removed with a high‐pass filter (128 s).

Image processing was done in SPM8. False alarms, correct rejections, and go trials were modeled separately with the standard hemodynamic response function (event duration 4000 ms from stimulus onset), along with six realignment parameters and white matter signal intensity as nuisance variables. Our primary construct of interest was activation associated with successful inhibitory control. For each participant, images that represented the hemodynamic response associated with CRs versus FAs (i.e., successful vs. failed inhibition) and CRs versus implicit baseline were computed. Comparing CR activity with FA activity holds the stimulus (nontarget, or “no‐go”) constant. In addition, an implicit baseline was used as opposed to target (i.e., “go”) trials because of the high frequency of target trials relative to the other event types (Devito et al., 2013).

At the group level, one‐sample *t*‐tests in SPM8 were used to detect activation associated with correct rejections (i.e., CRs vs. FAs, CRs vs. baseline). We performed a whole‐brain search at a family‐wise error (FWE) corrected threshold of *p *<* *.05 and a voxel extent >25; significant clusters in prefrontal and striatal areas (i.e., those associated with COMT functioning) were identified, and beta values were extracted using MarsBaR (Brett, Anton, Valabregue, & Poline, 2002) and imported into IBM SPSS Statistics v.22 (IBM Corp, 2013) for further analysis.

#### 2.4.1 Demographic, psychometric, and task performance measures

Fisher's exact tests or one‐way analysis of variance (ANOVA) were used to test for differences across genotype on demographic and psychometric variables (Table 1). Differences related to sex and genotype on Go/no‐go task measures (hit rate, hit reaction time, false alarm rate, false alarm reaction time, and *d*') were tested with a multivariate analysis of variance (MANOVA; means and standard deviations in Table 2).

#### 2.4.2 Neuroimaging measures

Two‐way ANOVAs (sex* *× genotype) were used to test main effects and the interaction of sex and genotype on brain activity, restricted to significant frontal and subcortical clusters from the one‐sample *t*‐tests of CRs versus FAs and CRs versus baseline (Table 3). Correction for testing multiple comparisons was applied using the Benjamini–Hochberg false discovery rate (FDR) procedure (Benjamini & Hochberg, 1995; *Q *=* *.05, *m *=* *8) for each of the three effects (i.e., main effect of genotype, main effect of sex, interaction of sex and genotype).

**Table 3:** Main effects of two go/no‐go task contrasts and two‐way ANOVA tests of genotype* *× sex

	*x*	*y*	*z*	*k*	*t*‐value	FWE *p*‐value	Main Effect of Genotype *F*(2, 59)	Main Effect of Sex *F*(1, 59)	Interaction *F*(2, 59)
CRs vs. FAs									
R. Putamen	20	18	−2	131	6.40	.001	***F *** **=** *** *** **5.83** ***p *** **=** *** *** **.005** a **partial η** ^**2**^ ** = .16**	*p *=* *.259	***F *** **=** *** *** **3.18** ***p *** **=** *** *** **.049partial η** ^**2**^ ** = .10**
L. Putamen	−14	16	−4	136	6.33	.001	***F *** **=** *** *** **5.76** ***p *** **=** *** *** **.005** a **partial η** ^**2**^ ** = .16**	*p *=* *.976	*p *=* *.422
CRs vs. Baseline									
B. Caudate	16	24	−6	789	7.90	<.001	*p *=* *.582	*p *=* *.245	***F *** **=** *** *** **3.93** ***p *** **=** *** *** **.025partial η** ^**2**^ ** = .12**
B. SMA/Mid Cingulate	−6	10	42	1093	7.24	<.001	*p *=* *.164	*p *=* *.310	*p *=* *.108
L. Pre‐/Postcentral Gyrus	−60	2	22	142	6.07	.002	*p *=* *.084	*p *=* *.096	*p *=* *.404
L. IFG/Operculum	−68	−8	4	31	6.01	.002	*p *=* *.126	***F *** **=** *** *** **5.03** ***p *** **=** *** *** **.029partial η** ^**2**^ ** = .08**	***F *** **=** *** *** **3.32** ***p *** **=** *** *** **.043partial η** ^**2**^ ** = .10**
R. OFC	36	50	−12	60	6.00	.002	*p *=* *.374	*p *=* *.124	*p *=* *.118
R. IFG/Insula	46	14	−2	42	5.85	.004	***F *** **=** *** *** **5.20** ***p *** **=** *** *** **.008** a **partial η** ^**2**^ ** = .15**	*p *=* *.363	*p *=* *.641

L, left; R, right; B, bilateral; k, cluster size; CR, correct rejection; FA, false alarm; SMA, supplementary motor area; IFG, inferior frontal gyrus; OFC, orbitofrontal cortex; η, eta.

Coordinates are in Montreal Neurological Institute (MNI) space. Results (from SPM8) are significant at family‐wise error rate (FWE) corrected *p *<* *.05 with a 25 voxel extent threshold; one peak voxel is reported per cluster.

a Main effects and/or interactions (from SPSS) significant at false discovery rate (FDR) corrected *p *<* *.05 (Benjamini–Hochberg procedure; *Q *=* *.05, *m *=* *8). A measure of effect size (partial η^2^) is given for effects (from SPSS) significant at *p *<* *.05, uncorrected (in bold).

## 3 Results

### 3.1 Psychometric variables

There were no significant genotype group differences, tested via one‐way ANOVAs or Fisher's exact tests, for participant sex (*p *=* *.940), age (*p *=* *.485), race^3^ (*p *=* *.124), parental history of AUD (ever: *p *=* *.377; in child's lifetime: *p *=* *.883), IQ (*p *=* *.897), externalizing symptomology (*p *=* *.154), internalizing symptomology (*p *=* *.102), DSM‐IV diagnoses (all *p*s = .476), or substance use (*p *=* *.554) (Table 1).

### 3.2 Go/no‐go task performance measures

Task performance measures (i.e., hit rate, false alarm rate, button press reaction times, *d*'; Table 2) were tested with a MANOVA with two factors (male vs. female; Met/Met vs. Val/Met vs. Val/Val). There was a significant effect of sex on task performance measures, *F*(5, 55)  = 2.53, *p *=* *.039; follow‐up tests revealed a significant effect of sex on false alarm rate, *F*(1, 59) = 9.81, *p *=* *.003 (males > females). There were also trend‐level effects of sex on *d*', *F*(1, 59) = 3.65, *p *=* *.061 (females > males) and hit reaction time, *F*(1, 59) = 3.12, *p *=* *.083 (females > males). There were no other significant effects.

### 3.3 Imaging results

#### 3.3.1 Main effects of successful inhibition

##### CRs versus FAs

**Figure 1:**
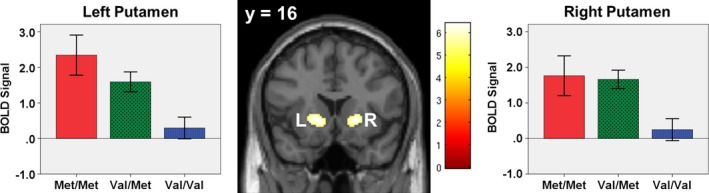
Correct Rejections versus False Alarms, Main Effect of Genotype. Whole‐brain main effects analysis of correct rejections versus false alarms showed activation in left and right putamen. These regions are significant at a family‐wise (FWE) corrected threshold of *p *<* *.05, with a 25 voxel extent. The color bar represents *t*‐values and the *y*‐coordinate is in Montreal Neurological Institute (MNI) space. Bar graphs depict significant main effects of genotype on mean cluster blood oxygenation level‐dependent (BOLD) signal. Error bars are ± 1 standard error. L = left; R = right. Coordinates and statistics can be found in Table 3

A one‐sample *t*‐test on the contrast of correct rejections (CRs) versus false alarms (FAs) showed significant activation in the right and left putamen (Fig. 1, Table 3).

##### CRs versus Baseline

The contrast of CRs versus baseline showed significant activation in several frontal and subcortical brain areas (Table 3). Specifically, significant clusters were found the bilateral caudate, bilateral supplementary motor area (SMA)/mid cingulate, left Pre‐/Postcentral gyrus, left inferior frontal gyrus (IFG)/operculum, right orbitofrontal cortex (OFC), and right IFG/insula.

#### 3.3.2 Effects of sex, genotype, and sex* *× genotype

**Figure 2:**
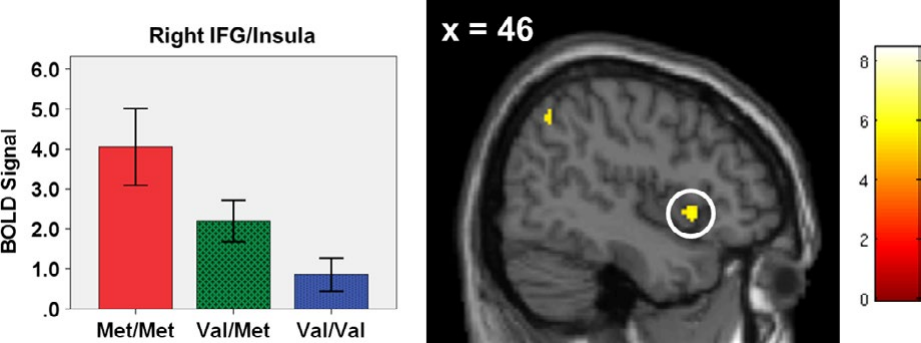
Correct Rejections versus Baseline, Main Effect of Genotype. One cluster from the whole‐brain main effects analysis of correct rejections versus baseline showed a significant main effect of genotype (right inferior frontal gyrus [IFG]/insula, circled). This region is significant at a family‐wise (FWE) corrected threshold of *p *<* *.05, with a 25 voxel extent. The color bar represents *t*‐values and the *y*‐coordinate is in Montreal Neurological Institute (MNI) space. The bar graph depicts a significant main effect of genotype on mean cluster blood oxygenation level‐dependent (BOLD) signal. Error bars are ± 1 standard error. Coordinates and statistics can be found in Table 3

Two‐way ANOVAs (sex* *× genotype) were performed in SPSS using significant clusters from the SPM one‐sample *t*‐tests of main effects of successful inhibition (Table 3). There was a significant main effect of genotype in right and left putamen (Fig. 1) and right IFG/insula (Fig. 2). These effects remained significant after controlling for testing multiple comparisons. Follow‐up tests revealed that in both the left and right putamen, both Met/Met homozygotes and Val/Met heterozygotes had significantly higher activity than Val/Val homozygotes. In right IFG/insula, Met/Met homozygotes had significantly higher activity than Val/Val homozygotes.

There was also a main effect of sex in the left IFG/operculum and a sex by genotype interaction in the right putamen, bilateral caudate, and left IFG/operculum. However, these findings did not pass correction for multiple comparisons.

#### 3.3.3 Correlations with task performance measures

In the full sample, activity in the right OFC was significantly negatively correlated with hit rate, *r*(65) = −.25, *p *=* *.048 and *d*', *r*(65) = −.28, *p *=* *.025. Activity in the right IFG/insula was also significantly negatively correlated with *d*', *r*(65) = −.26, *p *=* *.038. When testing correlations in males and females separately, activity in three regions was significantly negatively correlated with hit rate in females but not in males: left IFG/operculum, *r*(22) = −.53, *p *=* *.010; right OFC, *r*(22) = −.46, *p *=* *.031; and right IFG/insula, *r*(22) = −.50, *p *=* *.019. Also in females, there was a significant negative correlation between *d*' and activity in left IFG/operculum, *r*(22) = −.51, *p *=* *.016 as well as right IFG/insula, *r*(22) = −.44, *p *=* *.039. In males, there was a significant negative correlation between *d*' and activity in right OFC, *r*(43) = −.34, *p *=* *.023.

## 4 Discussion

This study investigated the association between the *COMT* Val^158^Met polymorphism and neural activity during response inhibition in male and female youth at high‐risk for SUD. During the successful inhibition of a prepotent motor response, a network of regions including the left and right putamen, bilateral caudate, and right IFG/insula were activated, consistent with prior reports of response inhibition circuitry (Buchsbaum, Greer, Chang, & Berman, 2005; Garavan, Ross, & Stein, 1999; Simmonds, Pekar, & Mostofsky, 2008). Three regions activated during successful inhibition showed a significant main effect of genotype—left and right putamen and right IFG/insula—with Met/Met homozygotes having higher activity than Val/Val homozygotes, contrary to hypotheses that Val/Val homozygotes would show the highest levels of brain activity. Also contrary to hypotheses that effects would be stronger in males, no significant interactions with sex were observed.

For the contrast of correct rejections versus false alarms (CRs vs. FAs), we found significant activation in the left and right putamen that was also significantly associated with *COMT* genotype. It has been suggested that greater activation of the putamen during successful compared with failed inhibition may reflect dopaminergic processes associated with trial‐and‐error learning (Holroyd & Coles, 2002; Stevens, Kiehl, Pearlson, & Calhoun, 2009). The right IFG/insula is a region classically associated with successful inhibitory control (e.g., Aron, Fletcher, Bullmore, Sahakian, & Robbins, 2003), and may act by exerting goal‐directed influences consistent with executive control of behavior (Stevens, Kiehl, Pearlson, & Calhoun, 2007).

Despite the associations among mesolimbic dopamine, inhibitory control, and risk for SUD, the effects of the *COMT* Val^158^Met polymorphism on brain activation during response inhibition have not been examined in high‐risk youth. The function of the COMT enzyme is to degrade dopamine, with the Met version of the polymorphism coding for the low activity enzyme and resulting in higher levels of dopamine. Our findings indicate that the highest BOLD activity during inhibitory control is exhibited by those with the highest dopamine levels (i.e., Met/Met homozygotes). Accordingly, lower levels of BOLD activity corresponded with lower levels of dopamine (Val/Val homozygotes). Considering there were no significant differences in task performance related to genotype, these findings can be interpreted as lower efficiency during successful inhibition in Met/Met individuals relative to the Val/Val homozygotes, with Val/Met heterozygotes displaying intermediate levels of efficiency. It is important to note, however, that the interpretation of lower efficiency in Met/Met participants is not specific to any one underlying mechanism (see Poldrack, 2015). Potential biological processes that could explain differential activation in the context of similar task performance between groups include performing different cognitive processes or neural computations. It is also possible that groups differ on neural computation intensity or timing.

The extant human and animal literature suggests that cognitive task performance and dopamine concentration follow an inverted U relationship (Arnsten & Goldman‐Rakic, 1998; Bilder, Volavka, Lachman, & Grace, 2004; Mattay et al., 2003), with too little or too much dopamine resulting in reduced cognitive functioning. The nature of this association with regard to *COMT* is well‐established in adults; it is the Met/Met genotype that lies at the top of the curve and is thus the optimal polymorphism (e.g., Egan et al., 2001). A study of healthy adult subjects in which the COMT enzyme was pharmacologically manipulated further supports this: Administration of tolcapone, a COMT enzyme inhibitor, improved executive functioning in subjects with the Val/Val genotype, but worsened performance in Met/Met subjects (Apud et al., 2007). However, there is also substantial evidence this relationship is not static throughout development. Dopaminergic concentrations increase in early‐ to mid‐adolescence before dropping throughout adulthood (reviewed in Spear, 2000), effectively shifting the location of the *COMT* genotypes on the inverted U from childhood to adolescence to adulthood (reviewed in Wahlstrom, Collins, White, & Luciana, 2010). Thus, the advantage belongs to Val/Met heterozygotes during adolescence (Wahlstrom et al., 2007) and, as we found here, Val/Val homozygotes in late childhood.

Here we found that Val/Val subjects had reduced brain activity during successful inhibitory control in the absence of genotype‐related task performance differences. Thinking more broadly about *COMT* and impulsivity in general, the literature—based primarily on adult samples—is not clear. Theoretically, the Val allele is thought to lead to weakened inhibitory control and a propensity to impulsivity by way of enhancing flexibility, whereas the Met allele is thought to enhance inhibitory control by dampening cortical noise (reviewed in Congdon & Canli, 2008). Boettiger et al. (2007) indeed found that Val/Val individuals demonstrated a more impulsive pattern of choice behavior than the other two genotypes on a temporal discounting task in healthy adults. On the other hand, also in a sample of healthy adults, Soeiro‐de‐Souza, Stanford, Bio, Machado‐Vieira, and Moreno (2013) reported that the nonplanning impulsiveness factor of the Barratt Impulsiveness Scale‐11 (Patton, Stanford, & Barratt, 1995) was higher in Met/Met subjects than in Val/Val subjects. Other studies have found this general pattern as well: DeYoung et al. (2010) and Biederman et al. (2008) both found that the Met allele was associated with ADHD symptoms. Finally, a recent study of male children and adolescents found significantly higher hyperactive‐impulsive and inattentive scores in Met/Met individuals (Perkovic et al., 2013). Indeed, the relationship between *COMT* and impulsivity is not clear‐cut, but is nonetheless essential for understanding how genetic variation, behavior such as inhibitory control and impulsivity, brain function, and substance use problems are related.

Disinhibitory psychopathologies, including substance use problems, are often comorbid with other disorders. Thus, these findings may have broader implications in terms of risk for a variety of other psychopathologies as well. In adults, *COMT* has been associated with prefrontal functioning and executive control in schizophrenia (Ehlis, Reif, Herrmann, Lesch, & Fallgatter, 2007), social cognition in bipolar disorder (Soeiro‐de‐Souza et al., 2012), as well as risk for obsessive‐compulsive disorder (Azzam & Mathews, 2003) and early onset major depressive disorder (Massat et al., 2005). As this study is part of a larger ongoing longitudinal project, it will be critical to follow these participants throughout their teens and twenties to examine links between the present results and the future development of disinhibitory and other psychopathologies, including substance use problems.

Contrary to recent work (White et al., 2014), we did not find significant sex* *× genotype interactions in brain regions associated with successful response inhibition after correcting for testing multiple comparisons. However, given the young age of the participants in this study (10.4 years) relative to those in the White et al. study (14.0 years), one possibility is that these interaction effects appear later in development, perhaps with the onset of puberty. Indeed, evidence suggests that estrogen may inhibit COMT activity (Schendzielorz, Rysa, Reenila, Raasmaja, & Mannisto, 2011; Xie, Ho, & Ramsden, 1999), an effect that may contribute to sex differences in *COMT* polymorphism effects in older samples. Another possibility is that given the limited distribution of participants across combinations of sex and genotype, our ability to detect sex* *× genotype interactions was artificially restricted. Future work in larger samples using a longitudinal design beginning in childhood and including measurement of hormonal concentrations will enable a more complete understanding of sex differences in *COMT* polymorphism effects on inhibitory control.

Regarding task performance measures, we did find that males had more false alarms than females, indicating a more impulsive responding style or a greater difficulty inhibiting a prepotent response, even prior to adolescence. There is mixed support in the literature for males being more impulsive than females (e.g., Campbell & Muncer, 2009). Those studies that have found sex differences related to impulsivity have focused primarily on risky or dangerous impulsivity rather than a more pure form that involves spontaneous action without the element of risk or danger. It is important to note here that the go/no‐go paradigm is not typically considered a measure of risky impulsivity. Still, these findings fit with the heightened prevalence of ADHD and other disinhibitory behavior in boys relative to girls (APA, 2000; Bauermeister et al., 2007). In the present sample, although just five of the eight participants diagnosed with ADHD were male, it is also possible that the heightened hyperactivity and impulsivity often seen in boys with ADHD relative to girls with ADHD (American Psychiatric Association, 2000; Gaub & Carlson, 1997; Hasson & Goldenring Fine, 2012) played some role. These findings are also interesting in light of the fact that use of various substances has frequently been found to be higher in boys than girls (Johnston, O'Malley, Miech, Bachman, & Schulenberg, 2015; SAMHSA, 2014) (though recent trends suggest less disparity than before). Again, this highlights the importance of following these participants throughout development to better understand the association between inhibitory control and later substance use.

We also found negative correlations between task measures and brain activity in the right OFC and IFG/insula in the full sample. Specifically, we found significant correlations with *d*' (a measure of sensitivity or discriminability) and hit rate, both of which reflect constructs that are distinct from inhibitory control. The correlations indicate that greater discriminability between go and no‐go stimuli was associated with less activation in the OFC and IFG/insula. In light of the main effect of genotype in the IFG/insula, with Val/Val subjects showing the lowest levels of activity, the negative correlation between discriminability and IFG/insula activity may be further evidence of a cognitive advantage for Val/Val homozygotes in childhood. Future studies with more subjects may find significant task performance differences by genotype to confirm this preliminary interpretation.

To our knowledge, this is the first fMRI study to examine the effects of the *COMT* Val^158^Met polymorphism on brain activity during response inhibition in children at high‐risk for SUD, but prior to their onset of significant substance use. The *COMT* Val^158^Met polymorphism was found to be associated with brain activity during response inhibition in high‐risk children aged 7–12, with Val/Val individuals showing the lowest levels of brain activity in three regions. This adds to a growing body of literature suggesting the importance of genetic variation in *COMT* in cognitive control and extends it to include high‐risk youth performing response inhibition. These results are also relevant for understanding how specific genes influence brain functioning related to SUD and other psychopathologies. It will be important for follow‐up studies to continue elucidating the pathway from dopamine‐related genes such as *COMT*, to inhibition‐related cognitive functioning, and finally to disinhibitory psychopathological outcomes, including substance abuse.

## Conflicts of Interest

The authors report no conflicts of interest.

## Notes

1 A subset of these participants (*n *=* *50) is the same as those from Hardee et al., 2014.

2 The number of runs excluded was as follows: Met/Met = 1 (female = 1); Val/Met = 6 (female = 2); Val/Val = 8 (female = 4). A 2 (sex) * *× 3 (genotype) analysis of variance (ANOVA) on the number of runs excluded was not significant for the main effect of sex, main effect of genotype, or interaction (all *p*s > .270). Sex* *× genotype ANOVAs on average motion, defined as the mean difference from one volume to the next (calculated for rotation and translation, respectively), were also not significant for the main effect of sex, main effect of genotype, or iteraction (all *p*s > .148).

3 See Supplemental Material for imaging analyses regarding race/ethnicity.
