# Supplementary figures and images for: Reduced brain activation during inhibitory control in children with COMT Val/Val genotype
Source: Brain Behav. 2016 Oct 5;6(12):e00577. doi: 10.1002/brb3.577 (PMC5167006; doi:10.1002/brb3.577)

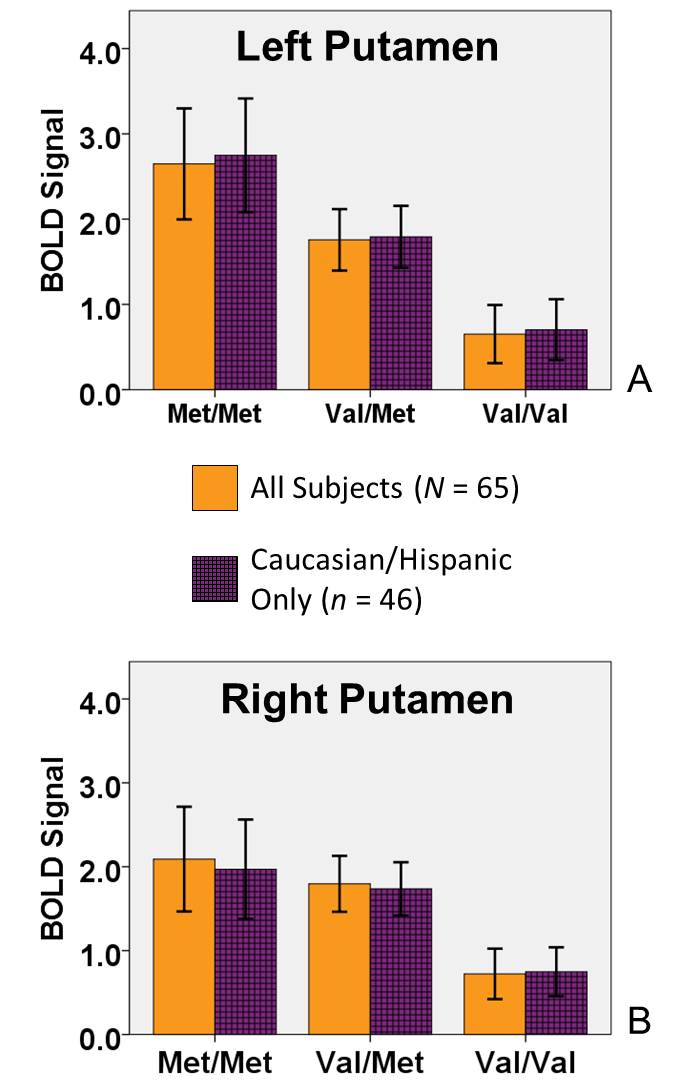

Supplement: Supplementary file 1 [file BRB3-6-e00577-s001.tif]
