# Supplementary material for: Reduced brain activation during inhibitory control in children with COMT Val/Val genotype
Source: Brain Behav. 2016 Oct 5;6(12):e00577. doi: 10.1002/brb3.577 (PMC5167006; doi:10.1002/brb3.577)
Supplement: Supplementary file 2 [file BRB3-6-e00577-s002.docx]

**Supplemental Material**

The association between genotype and race/ethnicity was examined. A 3 × 2 Fisher’s exact test of genotype (Met/Met; Val/Met; Val/Val) × race (Caucasian + Hispanic; African American + Biracial) was not significant (*p* = .405). As a validation of this nonsignificant association, a one-sample *t*-test on the fMRI contrast of correct rejections vs. false alarms was run again with only Caucasian and Hispanic subjects (*n* = 46). Results were substantively the same as in the full sample (*N* = 65). At a family-wise error (FWE) corrected threshold of *p* < .05, with a 25 voxel extent, two clusters were found: left putamen (*x* = -16, *y* = 16, *z* = -4; *k* = 112; *t*-value = 6.49) and right putamen (*x* = 20, *y* = 20, *z* = -2; *k* = 152; *t*-value = 7.11). These clusters were extracted using MarsBaR (Brett *et al.*, 2002) and two-way ANOVAs were performed in SPSS as in the full sample. See **Figure S1** for a comparison of results with the full sample (*N* = 65) and with Caucasian and Hispanic subjects only (*n* = 46) (effects of genotype shown).

**Figure S1 Legend.** Left and Right Putamen BOLD Signal in the Full Sample (*N* = 65) and in Caucasian and Hispanic Subjects (*n* = 46)

Results from the full sample and from Caucasian and Hispanic subjects only in the two *correct rejections vs. false alarms* clusters: left putamen (panel A) and right putamen (panel B). BOLD = blood oxygenation level-dependent.
